# Skin pH and buffering ability vary between two co-occurring semi-aquatic frog species

**DOI:** 10.1093/conphys/coaf037

**Published:** 2025-06-10

**Authors:** Einstein Nkwonta, Karen J Vanderwolf, Tyler Ambeau, Samuel Davison, April Kowalchuk-Reid, James E Paterson, Christina M Davy

**Affiliations:** Wildlife Research and Monitoring Section, Ontario Ministry of Natural Resources, 2140 East Bank Drive, Peterborough, ON, K9L 1Z8, Canada; Department of Biology, Trent University, 2140 East Bank Drive, Peterborough, ON, K9L 1Z8, Canada; Department of Biology, Trent University, 2140 East Bank Drive, Peterborough, ON, K9L 1Z8, Canada; Department of Biology, University of Waterloo, 200 University Avenue West, Waterloo, ON, N2L 3G1, Canada; Wildlife Research and Monitoring Section, Ontario Ministry of Natural Resources, 2140 East Bank Drive, Peterborough, ON, K9L 1Z8, Canada; Department of Biology, Carleton University, 1125 Colonel By Drive, Ottawa, ON Canada, K1S 5B6; Wildlife Research and Monitoring Section, Ontario Ministry of Natural Resources, 2140 East Bank Drive, Peterborough, ON, K9L 1Z8, Canada; Wildlife Research and Monitoring Section, Ontario Ministry of Natural Resources, 2140 East Bank Drive, Peterborough, ON, K9L 1Z8, Canada; Department of Biology, Trent University, 2140 East Bank Drive, Peterborough, ON, K9L 1Z8, Canada; Institute for Wetland and Waterfowl Research, Ducks Unlimited Canada, PO Box 1160, Stonewall, MB, R0C 2Z0, Canada; Wildlife Research and Monitoring Section, Ontario Ministry of Natural Resources, 2140 East Bank Drive, Peterborough, ON, K9L 1Z8, Canada; Department of Biology, Trent University, 2140 East Bank Drive, Peterborough, ON, K9L 1Z8, Canada; Department of Biology, Carleton University, 1125 Colonel By Drive, Ottawa, ON Canada, K1S 5B6

**Keywords:** Amphibians, pH, road salt, salinity, water chemistry

## Abstract

Amphibians face global declines linked to anthropogenic environmental change, including modifications to freshwater habitats. Human impacts on water chemistry, including acid rain and run-off of road salt into wetlands, may affect the physiology of amphibians with aquatic life stages. Specifically, water pH varies among freshwater habitats and affects amphibian development, behaviour, and physiology. For example, changes in skin pH affect the activity of enzymes on the skin, including those involved in antimicrobial functions. In this study, we explored the ability of free-ranging amphibians to maintain homeostasis across a range of naturally occurring water pH and salinity. We sampled two species of frogs at 19 wetlands around Peterborough, Ontario, measuring water pH, water salinity, and the skin pH of northern leopard frogs (*Lithobates pipiens*; *n* = 141) and green frogs (*Lithobates clamitans*; *n* = 329). We found that water pH increased with salinity, and was weakly related to the proportion of built-up habitat around wetlands. Frog skin pH was significantly associated with water pH, but both species showed a strong ability to buffer their skin pH across a range of conditions. On average, the ventral skin pH of *L. pipiens* increased by 0.37 units for each 1 unit increase in water pH, while skin pH of *L. clamitans* increased by 0.12. Specific responses to water chemistry differed between the two species: skin pH of *L. pipiens* varied with demographic group and body size, but skin pH of *L. clamitans* did not. As human effects on wetland habitats increase, these amphibians’ ability to buffer skin pH may provide some protection against anthropogenic changes in wetland water chemistry.

## Introduction

Anthropogenic threats to freshwater habitats include water pollution (e.g. roadway run-off containing salts, oil and lead) and acid rain (Muschack, 1990; Mahaney, 1994; Lefcort *et al.,* 1998; Welsh and Ollivier, 1998; Collins and Russell, 2009). Amphibian population declines attributed to acid rain and snowmelt in parts of Europe, Scandinavia and North America have stimulated research on the influence of environmental chemistry on amphibians (Pierce, 1985; Leuven *et al.,* 1986; Räsänen *et al.,* 2002). Amphibian skin is thin and permeable, which facilitates water uptake, ion transport, respiration and heat transfer (Varga *et al.,* 2019), but also leaves them vulnerable to desiccation (Duellman and Trueb, 1986). Chemical alterations to freshwater habitats of amphibians may also affect the physical and chemical properties of amphibian skin, limiting physiological function and potentially increasing susceptibility to cutaneous or systemic diseases (Varga *et al.,* 2019).

The pH of freshwater habitats such as ponds and wetlands generally falls between 6.5 and 9.0 pH, and is determined by complex relationships between carbon dioxide, hardness, photosynthesis and respiration (Pierce, 1987; EPA, 2012; USGS, 2013). However, anthropogenic influences such as the inputs of acid rain and wastewater can drive water pH beyond this range. (Gorham, 1998). Water pH may also be affected by the influx of salt from road de-icing (Kim and Koretsky, 2013).

Changes in water pH can affect amphibians by changing ion transport across the skin (skin pH). Low pH (<5 pH) depresses sodium uptake and increases sodium loss (Freda and Dunson, 1984, 1985; Meyer *et al.,* 2021). The resulting sodium loss can cause physical and behavioural abnormalities, slow growth rates, inhibit hatching, decrease sperm motility and kill amphibian embryos and larvae (Schlichter, 1981; Pierce, 1985; Green and Peloquin, 2008; Farquharson *et al.,* 2016; Meyer *et al.,* 2021). Amphibian larvae are generally more tolerant of acidity than embryos, and their tolerance increases as the larvae grow and develop into adults (Friedman *et al.,* 1967; Pierce *et al.,* 1984; Freda and Dunson, 1985; Pierce, 1985). The sensitivity of amphibian species to low pH varies widely both among and within species, and some frogs can tolerate water pH as low as 3.5 while others suffer severe sodium loss in water with pH 5 (Friedman *et al.,* 1967; Pierce, 1985; Freda, 1986; Meyer *et al.,* 2021). Shifts in water pH can also affect amphibians indirectly, because the solubility and toxicity of chemicals and heavy metals varies with water pH (Freda and McDonald, 1993; Vertucci and Corn, 1996; Edginton *et al.,* 2004; Ezemonye and Enuneku, 2005).

Environmental pH has implications for disease susceptibility and severity. Chytridiomycosis is a skin disease caused by the fungi *Batrachochytrium dendrobatidis* and *Batrachochytrium salamandrivorans,* which has resulted in severe population declines and even extinctions among amphibians (Scheele *et al.,* 2019). While *B. dendrobatidis* can grow *in vitro* at 4–8 pH (maximum growth at 6–7 pH), the activity of enzymes produced by the fungus varies with pH and i highest at 8 pH (Piotrowski *et al.,* 2004). The activity of amphibian antimicrobial skin peptides against fungi are also influenced by pH (Sheafor *et al.,* 2008,), and water pH influences the skin microbial community structure of frogs, which may further affect disease susceptibility (Krynak *et al.,* 2015).

Vertebrate skin pH is normally ~5–8 pH, but varies by species, season, sex, age and body part (Vanderwolf *et al.,* 2021) and with changes in environmental pH. Fish can maintain their skin pH at lower levels compared to water pH (Litwiller *et al.,* 2006,), and northern leopard frogs (*Lithobates pipiens*) tend to prevent lowering of skin surface pH <6 by releasing NaHCO_3_ from the skin (Friedman *et al.,* 1967). Understanding how tightly skin pH in amphibians is linked to environmental chemistry and how well amphibians can buffer their skin pH against environmental changes can help predict the susceptibility of amphibians to ongoing anthropogenic effects on freshwater habitats.

We explored this question in a heavily settled landscape in southern Ontario, Canada, where urbanization and the application of road salt to roads in the winter directly affect water chemistry in the habitat of semi-aquatic frogs such as *L. pipiens* and green frogs (*Lithobates clamitans*). To provide a foundation for future mechanistic studies on the physiological effects of these changes, our first objective was to characterize water chemistry in our study area by quantifying the association between water pH, salinity and land cover. We hypothesized that anthropogenic habitat surrounding wetlands contribute to alkalinization of wetland waters through road salt and other run-off. Our second objective was to explore the effects of water chemistry on frog skin pH, by (1) determining how frog skin pH correlates with water pH, time of year, sex, age class and body size; and (2) exploring interspecific variation in the response of frog skin pH to water pH and salinity. We hypothesized that frogs would have limited ability to maintain homeostasis by buffering their skin pH, predicting that skin pH would be tightly linked with water chemistry.

## Materials and Methods

### Data collection

We collected paired measurements of frog skin pH and water pH at 19 sites near Nogojiwanong (Peterborough), Ontario, Canada, on the traditional territory of the Michi Saagiig Anishnaabeg, which are lands covered by the Williams Treaty of 1923. Sites comprised of bogs, marshes, roadside ditches and stormwater drains. We sampled each site in June 2021 and again in July 2021, with an interval of 2–3 weeks between the first and second site visits.

We focused our sampling on *L. pipiens* and *L. clamitans* that co-occur in the study area. Both species are habitat generalists, exposing them to a range of conditions among various types of wetlands (Loder *et al.,* 2019). We aimed to capture at least 10 individuals of each species at each site. We caught frogs using dip nets with hoops of at least 46 cm, and mesh fine enough to prevent escape. We wore a new, sterile pair of nitrile gloves to catch and handle each frog. Prior to measurements, frogs were held in individual, 1-l plastic tubs filled with water collected from their specific capture location (within a few centimetres of the point of capture).

We identified each frog to species and determined sex by assessing the size of the tympanum, which is larger than the eye in male *L. clamitans*, and equal to or smaller than the eye in females. In *L. pipiens,* the tympanum of the male is smaller than the eye. We used a measuring tape to measure curved dorsal snout–vent length (the distance from the tip of the nares to the vent along the dorsal surface). We caught adult frogs at our first site visit. The second site visit overlapped with the emergence of recently metamorphosed individuals, and we captured adults and juveniles.

We measured the skin pH of each frog using the non-invasive pH meter (PH905; Courage and Khazaka Electronic GmbH, Mathias-Brüggen-Str. 9 150 829 Köln, Germany), which attaches to a multiprobe adapter system (MPA2; Courage and Khazaka Electronic). We quantified each individual’s dorsal and ventral torso skin pH, collecting three measurements from each body part and using the mean as the final value. The probe is very sensitive, and variation in the position of the probe relative to the skin can produce variation in the measured values. If repeated measurements of a body part differed by more than 0.2 pH, we retook measurements. We stored the end of the skin pH probe in KOH and washed it in distilled water between each set of measurements, as recommended by the manufacturer. We calibrated the pH probe once a week with 4 and 7 pH buffers, exceeding the manufacturer’s recommendation of calibration every 3 weeks. We also measured the pH and salinity of the water in which each frog was collected and held, using a Waterproof ExStik© pH meter (Extech Instruments) and Extech EC170 Salinity/Temperature meter, following the manufacturer’s instructions.

After measurements were completed, we placed each frog into a large bin with the other sampled frogs to avoid resampling previously captured individuals. We released all captured frogs at the end of each survey. We sterilized our gear (nets, boots, etc.) with a bleach solution between surveys, following the decontamination protocols recommended by the Canadian Herpetofauna Health Working Group (Canadian Herpetofauna Health Working Group, 2017) to avoid potential transmission of pathogens such as ranavirus and chytrid fungi among sites.

All capture and handling of the frogs described here was authorized by the Ontario Ministry of Natural Resources and Forestry, following a protocol approved by the Wildlife Animal Care Committee of the OMNRF. To test how human-impacted habitat affected water chemistry, we measured the proportion of built-up habitat within 200 m of a site’s coordinates, including agricultural fields, roads, pervious and impervious built-up areas using the Southern Ontario Land Resource Information System (Ontario Ministry of Natural Resources and Forestry, 2019).

### Statistical analysis

We analysed all data with R (R Core Team, 2020) and fit linear mixed-effects models with the *lme4* package (Bates *et al.,* 2015). We tested the main effects using Type III Wald F tests and Kenward–Roger estimates for degrees of freedom. We inspected the residuals of each model to check that they met assumptions of normality and heteroskedasticity.

We used a linear mixed-effects model to test how salinity, day of the year and the proportion of built-up habitat (fixed factors) predicted water pH (response variable). We included sampling site as a random effect to account for other variation among the sampling sites that was not captured in our predictor variables, which might affect water pH.

To test intra-individual variation in frog skin pH, we calculated Pearson’s correlation coefficient for ventral and dorsal skin pH. Upon finding a strong correlation in both *L. pipiens* (*r* = 0.80, *t* = 15.47, *df* = 138, *P* < 1.0 × 10^−15^) and *L. clamitans* (*r* = 0.79, *t* = 23.39, *df* = 336, *P* < 1.0 × 10^−15^), we proceeded with ventral skin pH for all subsequent analyses.

Next, we used a linear mixed-effects model to test how frogs’ ventral skin pH (response variable) was affected by three fixed effects: demographic group (juveniles, adult males, adult females), body size (curved dorsal length) and the interaction between demographic group and body size. We also included the sampling site as a random factor. We built separate models for *L. pipiens* and *L. clamitans*.

Finally, we used a linear mixed-effects model to test how a frog’s ventral skin pH (response variable) was affected by water pH, demographic group (juveniles, adult males, adult females), site visit (a proxy for seasonal changes in habitat) and sampling site (random factor). We built separate models for *L. pipiens* and *L. clamitans*. We used histograms of the difference between frog ventral skin pH and the water pH for each species to visualize species patterns in limits to buffering between skin and environmental (water) pH.

## Results

We captured 141 *L. pipiens* and 339 *L. clamitans* at 19 wetlands between 21 May and 19 July 2021. We captured 2–21 *L. pipiens* (mean = 11) and 10–24 *L. clamitans* per wetland (mean = 18). Water pH at the sites varied from 5.61 to 8.43 (mean = 7.32) and salinity varied from 0 to 0.315 parts per thousand (mean = 0.137 parts per thousand). *Lithobates pipiens* skin pH ranged from 5.88 to 8.02 (mean ± SE = 7.05 ± 0.04) and *L. clamitans* skin pH ranged from 6.06 to 8.54 (mean = 7.07 ± 0.02).

**Figure 1:**
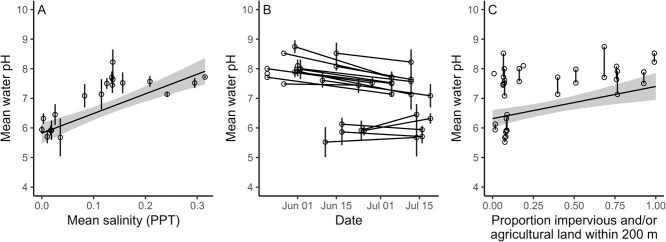
(**A**) Water pH increased significantly with salinity (parts per thousand; PPT) at 19 wetlands near Peterborough, Ontario, Canada (*n* = 255 water samples collected alongside captured frogs). Each point is the mean value from a visit to a wetland, and the vertical lines represent ±1 SE. (**B**) Mean water pH was not related to the sampling date. Each point is the mean value from a visit to a wetland, and the vertical lines represent ±1 SE. (**C**) Water pH increased significantly with the proportion of impervious and/or agricultural land within 200 m of the sample sites (but note the bimodal distribution of pH at sites sampled in wetlands farther from these built-up areas). In A and C, the solid trend lines show the model-predicted relationship (holding sampling date at the mean value) and the grey ribbons represent 95% CIs. In B and C, the black lines between pairs of points connect the two sampling events at each wetland.

Water pH increased significantly with increasing salinity (F = 33.38, *df* = 1, 16, *P* = 2.80 × 10^−05^, Fig. 1A). For every 0.1-part per thousand increase in salinity, the model predicted a pH increase of 0.73 (SE = 0.11). Water pH was not related to day of year (F = 210.41, *df* = 1, 1, *P* = 0.07, Fig. 1B). Water pH increased significantly with the proportion of impervious or agricultural land within 200 m (F = 9.86, *df* = 1, 16, *P* = 0.006, Fig. 1C). However, sites that were surrounded by more natural land cover fell into two groups. One group had lower pH than the wetlands in more developed areas, and these values drove the statistically significant trend estimated by the model. The other group had pH levels comparable to those measured near more built-up areas. We observed fewer *L. pipiens* at the most acidic sites, compared to the other surveyed wetlands. We captured 11 of 141 *L. pipiens* (7.8%), and 103 of 339 *L. clamitans* (30.4%) in water <7 pH.

**Figure 2:**
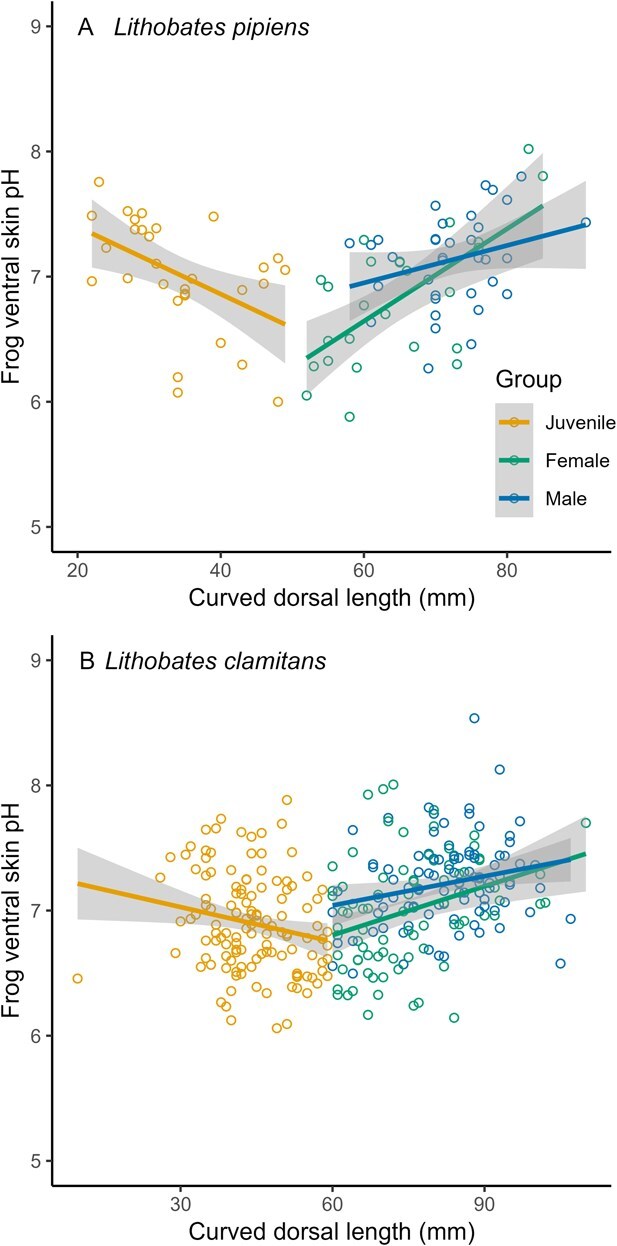
Sampling of frogs from 20 wetlands near Peterborough, Ontario, Canada, showed that ventral skin pH was significantly associated with body size in (**A**) northern leopard frogs (*L. pipiens*, *n* = 141). However, (**B**) this association was not significant in green frogs (*L. clamitans*, *n* = 339). Solid lines show the average effect of body size on skin pH predicted by a linear model, and the shaded areas represent 95% confidence intervals.

The ventral skin pH of *L. pipiens* varied with body size (F = 9.25, *df* = 1, 80, *P* = 0.003), demographic group (F = 4.57, *df* = 2, 80, *P* = 0.01), and the interaction between body size and demographic group (F = 3.53, *df* = 2, 81, *P* = 0.03, Fig. 2A). The relationship between ventral skin pH and body size was approximately flat for juveniles (slope = −0.01, 95% CI = −0.03–0.01), approximately flat in males (slope = 0.01, 95% CI = − 3.6 × 10^−3^–0.03), and positive in females (slope = 0.02, 95% CI = 0.01–0.04; all slope estimates are pH units/mm curved dorsal length). Ventral skin pH of *L*. *clamitans* did not vary with body size (F = 0.84, *df* = 1, 293, *P* = 0.36), demographic group (F = 1.17, *df* = 2, 290, *P* = 0.31), or the interaction between body size and demographic group (F = 1.22, *df* = 2, 289, *P* = 0.30, Fig. 2B).

**Figure 3:**
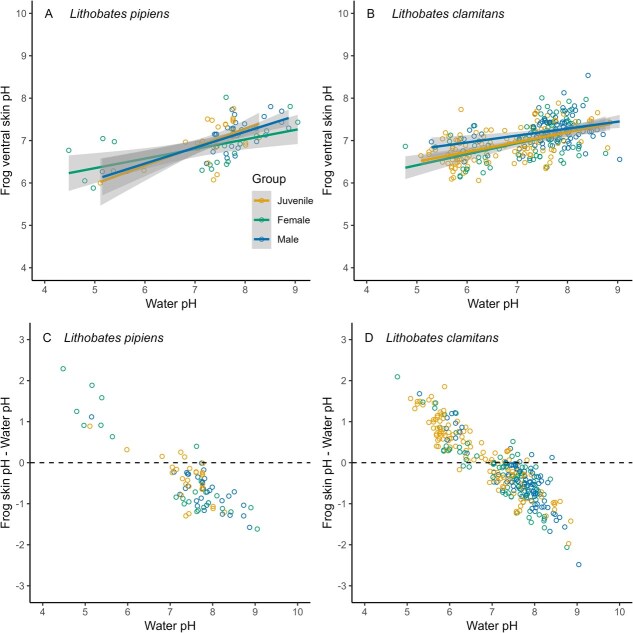
Frog ventral skin pH increased with water pH in (**A**) northern leopard frogs (*L. pipiens*, *n* = 141) and (**B**) green frogs (*L. clamitans*, *n* = 339) sampled at 19 wetlands near Peterborough, ON, Canada. The line is the predicted value from a linear model and the shaded areas represent 95% confidence intervals. Although these relationships were statistically significant, both *L. pipiens* (**C**) and *L. clamitans* (**D**) showed a remarkable ability to buffer their skin pH as water pH varied.

Ventral skin pH of *L. pipiens* increased with water pH (F = 17.62, *df* = 1, 34, *P* = 1.8 × 10^−4^), as did skin pH of *L. clamitans* (F = 8.68, *df* = 1, 97, *P* = 4.0 × 10^−3^). However, *L. clamitans* were better able to buffer their skin pH against changes in water pH. The model estimated that for a 1-unit increase in water pH, the ventral skin pH of *L. pipiens* increased by 0.37 (SE = 0.08), while the ventral skin pH of *L. clamitans* increased by only 0.12 (SE = 0.04). Overall, the difference between measured ventral skin pH and water pH varied from −1.64 to 2.29 (mean = −0.55 ± 0.06) in *L. pipiens* and from −2.48 to 2.09 (mean = −0.17 ± 0.04) in *L. clamitans* (Fig. 3A and B). The strongest association between frog skin pH and water pH (i.e. skin pH within 0.2 of water pH) occurred when water pH was a mean of 7.5 ± 0.4 SD for *L. pipiens*, and 7.3 ± 0.5 for *L. clamitans*. Despite the general, significant relationship between skin and water pH, this relationship was not 1:1. Instead, both species of frogs demonstrated an ability to buffer their skin pH to some extent when immersed in more acidic or alkaline wetlands (Fig. 3C and D).

## Discussion

Our analyses revealed that although frog skin pH is affected by water pH, both *L. pipiens* and *L. clamitans* have a remarkable ability to buffer their skin pH across a range of conditions to maintain homeostasis (Fig. 3C and D). The greater effect of water pH on skin pH in *L. pipiens,* and the lower capture rates of *L. pipiens* at more acidic sites, suggest that this species may be more vulnerable than *L. clamitans* in particularly acidic or alkaline environments. Water pH varied with land cover modification (Fig. 1), and likely with the input of road salt to road-adjacent wetlands, emphasizing how human effects on the landscape can impact wetland amphibians. However, while salinization of wetlands can create physiological challenges for amphibians (Karraker *et al.,* 2008), our results do not suggest that addition of road salt is causing water pH in our study area to exceed a tolerable range for frogs.

Demographic group (sex and age) were not significantly associated with skin pH in either species, and although the skin pH of *L. pipiens* increased with body size in adult females, this relationship was relatively weak. Given the relationship between water pH and ventral skin pH, we speculate that this difference may reflect sex-biased habitat use in *L. pipiens* (but see Blomquist and Hunter Jr, 2009)*.* Further data are required to test this speculation. However, our general measurements aligned with previous reports on skin pH in amphibians. Skin secretions of *L. pipiens* generally had a pH of 7.3–7.8 (max 8.6; Friedman *et al.,* 1967), slightly higher than the mean skin pH we measured. In frogs, diffusion of CO_2_ via the epidermis leads to acidification, and glandular secretions leads to alkalinization (Friedman *et al.,* 1967).

The ability to maintain optimal skin pH has consequences for basic physiological processes, such as the active transport of ions. In isolated frog skin (*Rana temporaria*), active Na + transport sharply diminishes when the pH at the epidermis of the skin falls <6 (Schoffeniels, 1955). Most frogs we measured had a skin pH > 6, even for frogs we caught in water pH < 6. Some frog species can tolerate water pH as low as 3.5, although the mechanisms behind this are unknown (Meyer *et al.,* 2021). Regardless of the tolerance range, salt balance and ion transport are important functions for amphibians both as aquatic larvae and as semi-aquatic adults. *Lithobates pipiens* were less frequently observed (e.g. apparently less abundant) at the more acidic sites, (Fig. 3; see also Vatnick *et al.,* 1999). This may imply these sites are approaching the threshold of tolerance for *L. pipiens.* Conversely, the greater ability of *L. clamitans* to buffer skin pH against a range of conditions may make them more tolerant of human activities that modify freshwater chemistry.

Alongside maintaining electrolyte balance, skin pH is associated with disease susceptibility in frogs. Skin has multiple defences against pathogens, including the production of antimicrobial skin peptides. When a tiger salamander (*Ambystoma tigrinum*) skin peptide preparation with an initial pH of 4.0 was combined with a Tris buffer of 7.4 pH, activity against the skin pathogen *B. dendrobatidis* was reduced >6.4 pH and nearly eliminated at 6.7 pH, suggesting these peptides are most active at low pH (Sheafor *et al.,* 2008). It is unclear if this pattern is true in frogs, and most frogs in our study had skin pH > 6.7. Potentially, skin peptides in our study species have different optimal pH compared to tiger salamanders. Pathogen loads were positively, weakly correlated with ventral skin pH in mountain yellow-legged frogs infected with *B. dendrobatidis*, suggesting frogs with more acidic skin may be more disease-resistant (Woodhams *et al.,* 2012). Changes in pH may affect another component of innate skin defences, the skin microbiome. A change in mean water pH from 7 to 6 caused a significant shift in the larval skin microbial community of American bullfrogs (*Lithobates catesbeiana*), which may affect disease susceptibility (Krynak *et al.,* 2015). Future research should investigate frog skin defences against pathogens over a range of pH conditions to test how water and skin pH affect interactions with pathogens.

We found that human impacts on water chemistry can impact the skin pH of free-ranging frogs. Although the association between land cover modification and water pH in our study was unclear, our most modified sites were still relatively functional wetlands. It would be interesting to extend this line of research by also sampling in heavily urbanized areas. While the frogs were able to buffer skin pH against a range of water pH in our study sites, this ability must have a limit. Skin pH can affect immune function and other physiological responses (Woodhams *et al.,* 2012; Krynak *et al.,* 2015), and human activity, wetland water chemistry and frog skin pH are all linked. In extreme cases, acidifying wetlands, such as from acid rain (Gorham *et al.,* 1984, p. 198; Gorham, 1998), or alkalinizing wetlands, such as from de-icing salt run-off (Kim and Koretsky, 2013), may move wetlands out of the range of pH in which some frog species can successfully breed and thrive. Therefore, ensuring that wetland water chemistry remains within tolerable limits for amphibians may help to maintain the diversity and abundance of frogs within them.

Our description of interspecific differences in the response of frog skin pH to water pH highlights the importance of multi-species monitoring approaches to predict the physiological effects of changing environmental chemistry. Our study species responded differently to wetland water pH, illustrating that reliance on single-indicator species may mask effects of environmental change in wetland communities. Our descriptive study was not designed to identify the mechanisms determining amphibian skin pH, but we demonstrated that baseline data on skin pH in wildlife provides a non-harmful and easily replicated biomarker for environmental monitoring. As chemical inputs from acid rain, road salt run-off and mining activities rapidly alter wetland water chemistry (Nordstrom *et al.,* 2015), understanding how resident amphibians are likely to respond can improve our understanding of potential future trends in biodiversity.

## Data Availability

The datasets generated and analysed during the current study are available in the Open Science Framework repository: https://osf.io/5jruf/.
